# Biocompatibility of Polymer and Ceramic CAD/CAM Materials with Human Gingival Fibroblasts (HGFs)

**DOI:** 10.3390/polym11091446

**Published:** 2019-09-03

**Authors:** María Rizo-Gorrita, Cristina Herráez-Galindo, Daniel Torres-Lagares, María-Ángeles Serrera-Figallo, José-Luis Gutiérre-Pérez

**Affiliations:** Department of Oral Surgery, College of Dentistry, Seville University, Calle de Avicena, s/n, 41009 Seville, Spain (M.R.-G.) (C.H.-G.) (D.T.-L.) (J.-L.G.-P.)

**Keywords:** polymethyl methacrylate (PMMA), silicates/chemistry, CAD-CAM, materials testing, biocompatible materials/chemistry, fibroblasts/cytology, cell survival, collagen type I

## Abstract

Four polymer and ceramic computer-aided design/computer-aided manufacturing (CAD/CAM) materials from different manufacturers (VITA CAD-Temp (polymethyl methacrylate, PMMA), Celtra Duo (zirconia-reinforced lithium silicate ceramic, ZLS), IPS e.max CAD (lithium disilicate (LS_2_)), and VITA YZ (yttrium-tetragonal zirconia polycrystal, Y-TZP)) were tested to evaluate the cytotoxic effects and collagen type I secretions on human gingival fibroblasts (HGFs). A total of 160 disc-shaped samples (Ø: 10 ± 2 mm; h: 2 mm) were milled from commercial blanks and blocks. Direct-contact cytotoxicity assays were evaluated at 24, 48, and 72 h, and collagen type I (COL1) secretions were analysed by cell-based ELISA at 24 and 72 h. Both experiments revealed statistically significant differences (*p* < 0.05). At 24 and 48 h of contact, cytotoxic potential was observed for all materials. Later, at 72 h, all groups reached biologically acceptable levels. LS_2_ showed the best results regarding cell viability and collagen secretion in all of the time evaluations, while Y-TZP and ZLS revealed intermediate results, and PMMA exhibited the lowest values in both experiments. At 72 h, all groups showed sharp decreases in COL1 secretion regarding the 24-h values. According to the results obtained and the limitations of the present in vitro study, it may be concluded that the ceramic materials revealed a better cell response than the polymers. Nevertheless, further studies are needed to consolidate these findings and thus extrapolate the results into clinical practice.

## 1. Introduction

Once implant osseointegration is achieved, a transepitelial abutment is placed for the soft tissue remodelling process [1]. The abutment material should be as similar as possible to the natural tissues in order to create a hermetic barrier [2] between the gingiva and abutment to achieve cellular proliferation and protect the underlying implant These materials must be biocompatible [2,3,4,5,6,7,8,9,10] because dental materials are always in contact with soft or bone tissue [6,11].

The study materials were milled using a computer-aided design/computer-aided manufacturing (CAD/CAM) system. This technology was developed in 1985 [12] and has improved modern dentistry, introducing new material designs. CAD/CAM offers a wide variety of advantages, such as reducing production time and achieving mimetic and perfectly adapted structures [2,6,13,14,15,16,17]. 

Zirconia is one of the most used CAD/CAM materials, but it suffers from expansion during cooling after sintering. This volumetric change is associated with the transformation from the tetragonal to monoclinic phase and can promote the propagation of cracks in its structure. The addition of a stabilizing agent, such as yttrium oxide (Y_2_O_3_), reinforces the zirconium oxide and prevents this transformation phase and the propagation of cracks. Adding 3–6% of yttrium to zirconia leads to the formation of yttrium-tetragonal zirconia polycrystal (Y-TZP) and is the type of zirconia most commonly used in dentistry [18,19,20,21]. 

Pre-sintered zirconia is usually employed to more easily handle the soft material and prevent the transformation phase induced by the milling of CAD/CAM materials, which are more susceptible to forming cracks on their structure [22]. Nevertheless, this material exhibits good mechanical properties, largely due to the particle size of the structure (0.2–0.5 µm), which helps to maintain the stable tetragonal phase. It presents a high flexural strength (900–1200 MPa), fracture resistance (7–10 MPa m^1/2^), and elasticity modulus (210 GPa). It is increasingly considered to be the alternative to titanium for aesthetic dental implant abutments in final restorations. 

Other ceramic materials are also widely used in dentistry [23,24], such as lithium disilicate (Li_2_Si_2_O_5_) glass ceramic. This material has been used for CAD/CAM manufacture since 2006 under the name IPS e.max CAD^®^ and has diverse indications (e.g., crowns, inlays, onlays, implant crowns, and veneers) because of its aesthetic properties and mechanical strength [2,6,17,23,24,25,26]. It is commercialised as pre-crystallised blue blocks, which have a metasilicate and lithium disilicate nucleus. This nucleus has good mechanical properties (flexural force of 130 MPa), which increase up to 360 MPa during the crystallisation process (sintering at 850 °C for 20–25 min), according to the manufacturer. Metasilicate is dissolved and leaves lithium disilicate crystals that can be coloured during the glazing process [2,6,13,15,23,24,25,27,28,29]. The final mechanical properties are a fracture resistance of 2.25 MPa m^1/2^, flexural strength of 360 MPa, and elastic modulus of 95 GPa [30].

A new material has emerged to satisfy the need for a material with the good mechanical properties of zirconia and the good aesthetic properties of lithium disilicate (LS_2_) [31]. This material is made by combining a vitreous matrix of lithium silicate and a crystal nucleus of zirconia, leading to the formation of zirconia-reinforced lithium silicate ceramic (ZLS). It was developed in 2013 by two manufacturers: Degudent and VITA Zahnfabrik at the Institute for Silicate Research in Germany. One of the commercialised materials is Celtra^®^ Duo and has a fast CAD/CAM manufacturing process and better optical and mechanical properties than LS_2_ [32,33,34]. It has a high fracture resistance of 2 MPa m^−1^, elastic modulus of 70 GPa, and flexural strength of 200 MPa [35,36,37], and it is used for single anterior and posterior crowns, inlays, onlays, and veneers [37].

Recently, CAD/CAM materials were developed for temporary restorations, one of which is poly(methyl methacrylate) (PMMA), which is a synthetic polymer material that has been used in dentistry since 1930 for orthodontics, removable prostheses, and splint manufacturing [38]. Its colour stability, resistance, and ease of preparation make it an ideal provisional material [38,39,40,41,42]. Interim restorations are an essential part of the process, especially in fixed implant prosthesis treatment [38,42], preventing bacterial contamination and improving abutment health [38]. Vita CAD-Temp^®^ was introduced to the market in 2005 as chemically improved polymer CAD/CAM blanks [43]. This PMMA-based polymer material is pre-polymerised before market distribution blocks. This reduces time and cost in the laboratory, eliminates polymerisation shrinkage [44], reduces clinical chairside, and unpleasant smells because the material is not mixed manually or in a cartridge, and it improves outcomes in terms of a better marginal fit and strength [12,38,42]. It is one of the most used temporary materials before the placement of the final restoration [38,45,46] and is also used in surgical guide manufacturing. It has good mechanical properties with a high elastic modulus (2800 MPa) and flexural resistance (>80 MPa), which makes it a long-term provisional material [39,47].

The aim of this in vitro study was to compare the early response of human gingival fibroblasts of four dental materials relative to biocompatibility and collagen secretion. To this end, we conducted an MTT cytotoxicity test, which measures cellular viability through mitochondria metabolism, and an enzyme-linked immunosorbent assay to measure collagen type I secretion.

To our knowledge, there are no other publications that compare these four materials in terms of their biocompatibility and COL1 secretion assays. Three publications compare some of these materials separately [13,17,48].

The null hypothesis was that the all-ceramic (Vita YZ^®^ T, ZLS Celtra^®^ Duo and IPS e.max^®^ CAD) and interim materials (Vita CAD Temp^®^) do not significantly influence HGF viability and collagen type I secretion.

## 2. Materials and Methods 

### 2.1. Sample Preparation

Four CAD/CAM materials groups were used: Vita CAD-Temp^®^ (VITA Zahnfabrik, Bad Säckingen, Germany), IPS e.max^®^ CAD (Ivoclar Vivadent, Schaan, Liechtenstein), VITA YZ^®^ T (Vita Zahnfabrik, Bad Säckingen, Germany), and Celtra^®^ Duo (Degudent GmbH, Hanau-Wolfgang, Germany); the manufacturers’ details are summarised in Table 1.

**Figure 1:**
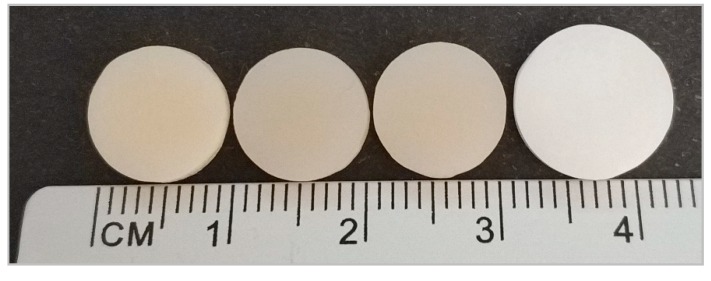
Materials (from left to right): PMMA, ZLS, LS_2_, and Y-TZP.

One hundred and sixty disc-shaped specimens (10 ± 2 mm in diameter and 2 mm in width) were milled from commercial blanks and blocks, as can be seen in Figure 1.

Discs were milled using InLab MC XL^®^ (Sirona, Bensheim, Germany) and Software InLab SW 16.1 (Sirona, Bensheim, Germany). This system works at 42,000 rpm and 320 VA and is irrigated with water and Dentatec (Sirona, Bensheim, Germany); the results obtained have high precision (tolerance value of ± 25µm). The milling process was made with the following drills: Step Bur 12S and Cylinder Pointed Bur 12S (PMMA blocks), Step Bur 12 and 12S, Cylinder Pointed Bur 12 and 12S (ZLS blocks), Step Bur 12S, 12 and 20, Cylinder Pointed Bur 12S, 20 and 12EF (LS_2_ blocks), and Step Bur 20, Cylinder Pointed Bur 20, Shaper 25 RZ and Finisher 10 (Y-TZP blanks, dry-milling).

Once the milling process was finished, lithium disilicate (LS_2_) was crystallised in Programat^®^ P700 (Ivoclar Vivadent, Schaan, Liechtenstein), a ceramic furnace that works in vacuum conditions at a temperature range between 840 and 850 °C for 20–30 min. Furthermore, the zirconia (Y-TZP) sintering process was performed in a VITA ZYRCOMAT 6000 MS furnace (VITA Zahnfabrik, Bad Säckingen, Germany) with the YT Universal program at 1530 °C for 4 h and 40 min.

Zirconia-reinforced lithium silicate (ZLS) blocks are commercialised in a fully sintered state with the final restoration shade; it is unnecessary to carry out additional sintering, unless better mechanical properties are demanded. This is a valid alternative to LS_2_ when high aesthetic quality and time savings are required. PMMA is a polymer composite, so there is no need for a sintering or crystallisation process, as indicated by the manufacturer.

**Figure 2:**
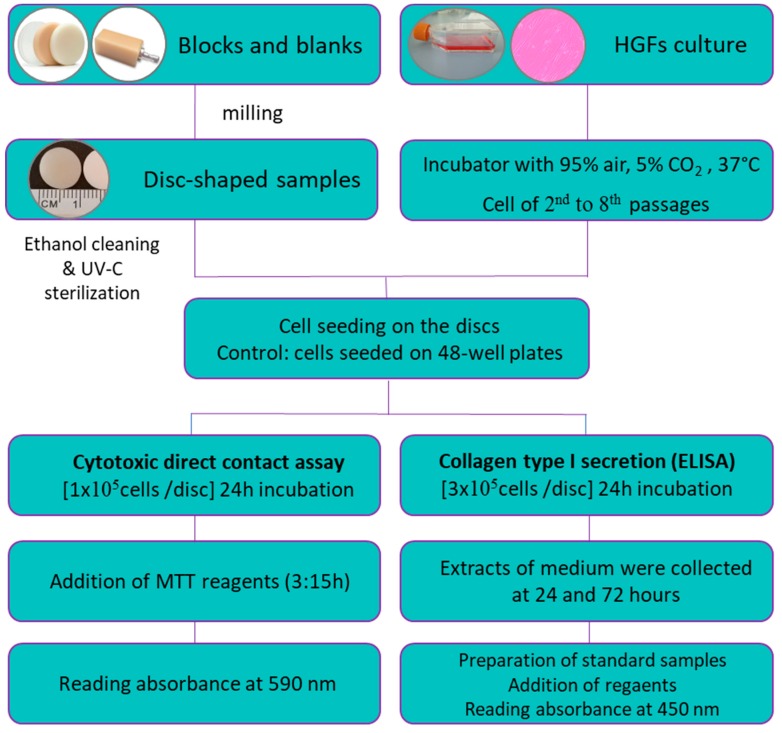
Diagram of the study design.

When the milling only (PMMA and ZLS) and the milling and sintering (Y-TZP and LS_2_) processes were finished, we obtained discs of 10 ± 2 mm diameter and 2 mm height (final dimensions of all discs). Discs were then cleaned by immersion with absolute ethanol and sterilised with short-wavelength (200–280 nm) UV-C exposure for 30 min on each side inside a laminar flow workstation. Next, discs were placed on sterile 48-well plates and were used as cell seeding substrates for both experiments (Figure 2).

### 2.2. Cell Culture

Human gingival fibroblasts (Innoprot, Bizkaia, Spain) were cultured in tissue flasks of polystyrene in a CO_2_ incubator in a Nuaire US Autoflow NU-4750-E (Nuaire, Plymouth, Minnesota, USA) at 37 °C in humidified 5% carbon dioxide (CO_2_) with a 95% air atmosphere for 1–2 weeks. Dulbecco’s modified Eagle medium (DMEM, Biowest, Nuaillé, France) was supplemented with 10% FBS (Biowest, Nuaillé, France) and 1% glutamine–penicillin–streptomycin (Biowest, Nuaillé, France). The medium was changed every 48 h, and the cells were subcultured regularly upon reaching 80% confluence. Later, the cells were passaged after trypsinization using 0.25% trypsin (Biowest, Nuaillé, France) and Dulbecco’s phosphate-buffered saline without calcium and magnesium (DPBS, Lonza, Basel, Switzerland), which was previously tempered in a water bath at 37 °C. Cellular growth, adhesion, and proliferation were monitored with an Olympus CKX41SF2 microscope (Olympus, Shinjuku-ku, Tokyo, Japan). Cultured HGFs from the second to eighth passages were used for the experiments.

### 2.3. Cytotoxicity Assay

With the purpose of evaluating the cytotoxicity of the materials, a cell viability assay was carried out at 24, 48, and 72 h. The protocol used is based on measurements of the viability of cells through metabolic activity in a colorimetric test.

MTT (3-(4,5-dimethylthiazol-2-yl)-2,5-diphenyltetrazoliumbromid) is a yellow-coloured tetrazolium water-soluble salt that is metabolically reduced by mitochondrial succinate dehydrogenase (SDH) from viable cells, produces formazan products (blue-violet salt), cannot cross plasmatic membranes, and accumulates in the cells [13,48,49,50,51]. The number of viable cells is correlated with the colour intensity determined by photometric measurements when formazan is dissolved in alcohol. The MTT cell proliferation assay (ab211091 kit, Abcam, Cambridge, UK) was performed by a direct-contact method according to ISO 10993-5:2009. This standard establishes that a material has a cytotoxic potential if cell viability is reduced below 70% [52].

Reaching 80% confluence, the cells were removed from culture flasks by enzymatic digestion Trypsin/EDTA 0.25% and centrifuged using Allegra™ X-22R Centrifuge (Bekman Coulter, Indianapolis, Indiana, USA) at 200 g for 3 min. A total of 96 discs was used for this experiment. Four discs were used for each material and evaluation time (24, 48, and 72 h) and were placed in 48-well plates. The same number of wells were used for the controls and blanks. Fibroblasts were seeded at a concentration of 1 × 10^5^ cells/disc in 500 µL of MEM (Sigma-Aldrich, St. Luis, MO, USA). The same cell concentration was cultured on empty 48-well plates as a control group. Wells containing only MEM were used as blanks. MEM was used without FBS or phenol red to avoid the overlapping of serum proteins and MTT absorbance.

At determined evaluation times, the medium was removed and replaced by MEM and MTT reagent during 3 h of incubation. Next, the formation of formazan crystals was checked under an inverted microscope and a dissolvent reagent was added. Plates were shaken for 15 min, and 250 µl of each well was placed in a 96-microwell plate. The optical density (OD) of the resulting solution was measured with a Synergy HT microplate reader (Biotek, Winooski, VT, USA) and Gen5™ Data Analysis Software (Winooski, VT, USA) at 590 nm. The mean absorbance values (samples and controls) were corrected for the mean absorbance of the blanks.

Cell viability was calculated as a percentage in relation to the control group, taken as 100% with the following formula: % viability = [(Sample absorbance – Blank absorbance) / (Control absorbance – Blank absorbance)] × 100.

### 2.4. Type I Collagen Secretion (ELISA)

To determinate the secretion of collagen type I into the medium by HGFs seeded on the discs, a double-antibody sandwich ELISA assay (ELISA Kit MyBioSource, San Diego, CA, USA) was performed. After 24 h and 72 h of incubation, the supernatant was collected, centrifuged, and analysed by an enzyme-linked immunosorbent assay. A total of 64 discs were used, with four discs per material and time evaluation. The cells were seeded at a density of 3 × 10^5^ cells/disc in 500 µl of MEM. MEM was used for the same reasons as those stated above. Discs were placed in 48-well plates for the experiment.

Briefly, dilutions were prepared in a dilution buffer. On an antibody-precoated 96-microwell plate, 100 µl of dilutions and 100 µl supernatants were added to each well. Collagen was detected by the human COL1 monoclonal antibody. Next, the biotin labelling antibody was added and washed with a washing buffer. Later, avidin–horseradish peroxidase conjugates were added to the wells; the plate was washed to remove the unbound enzyme-labelled antibodies. A TMB substrate was used to colour the peroxidase catalyst blue, which turns yellow upon reaction to sulfuric acid. The plates were read at 450 nm with a Synergy HT microplate reader and Gen5™ Data Analysis Software. The mean absorbance values (samples and controls) were corrected for the mean absorbance of the blanks. A standard curve was assessed to interpolate optical density (OD) values to the concentrations of collagen type I (ng/mL).

### 2.5. Statistical Analysis

The comparison of the groups for each of the analysed variables was made using IBM SPSS Statistics 24.0 software (International Business Machines Corp., New York, NY, USA). The Kolmogorov–Smirnov test was performed to verify normal distribution. The homogeneity equality of variance was checked using Levene’s test. One-way analysis of variance (ANOVA) was calculated to assess the statistical significance of differences in cell viability and collagen type I secretion. Post hoc comparisons were undertaken with Bonferroni and Games–Howell tests for equal or unequal variances, respectively. The level of significance was set at *p* < 0.05. Results were reported as a mean ± standard deviation (SD).

## 3. Results

### 3.1. Cytotoxicity of CAD/CAM Materials

**Figure 3:**
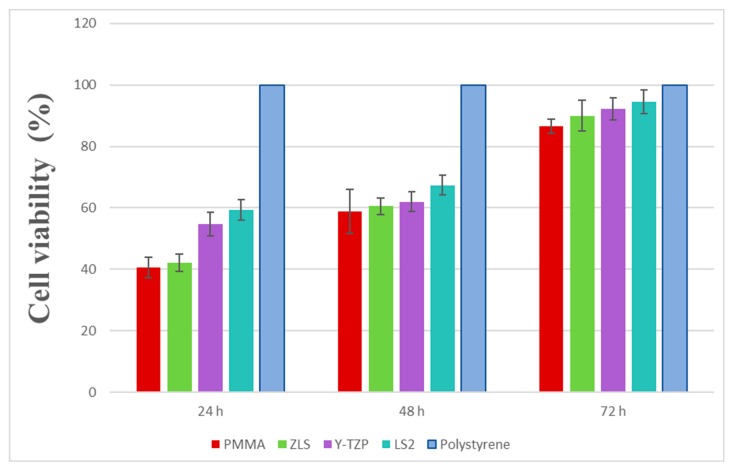
Cell viability percentage at 24, 48, and 72 h using MTT assay. The data are expressed as the mean values ± standard deviation.

In order to evaluate the cytotoxicity of the materials, cell viability was measured by a direct-contact MTT assay at three evaluation times (24 h, 48 h, and 72 h). The results revealed a significant and linear effect of time (*p* < 0.01) and type of material (*p* < 0.05) on the cell viability. Cytotoxicity decreased over time (Figure 3). Significant differences among groups were shown at 24 h (*p* < 0.01). LS_2_ revealed the highest viability (59.46% ± 3.32%) and PMMA the lowest (40.65% ± 3.32%); both were compared to the other materials (Y-TZP (54.74% ± 3.90%) and ZLS (42.20% ± 2.74%)). There were significant differences between all groups (*p* < 0.02), except between ZLS and PMMA (*p* > 0.05).

The same group distribution was seen at 48 h. There was an increase in the cell viability in all groups, with significant differences between them at 48 h (*p* < 0.05). LS_2_ showed the highest viability (67.35% ± 7.20%), and PMMA showed the lowest (58.85% ± 3.18%), compared with the LS_2_, Y-TZP (62.04% ± 3.26%) and ZLS (60.51% ± 2.69%) groups, but there were only significant differences when comparing LS_2_ with ZLS (*p* = 0.02) and LS_2_ with PMMA (*p* < 0.01).

At 72 h, all materials showed the highest viability between the three points of time evaluation. The group with the highest cell viability was LS_2_ (94.52% ± 2.30%), and PMMA showed the lowest (86.62% ± 3.75%). The statistically significant differences seen at 48 h remained at 72 h between LS_2_ and ZLS (*p* = 0.01) and LS_2_ with PMMA (*p* < 0.01). There was also a significant difference between Y-TZP and PMMA (*p* < 0.01).

### 3.2. Evaluation of Collagen Type I Secretion (ELISA)

**Figure 4:**
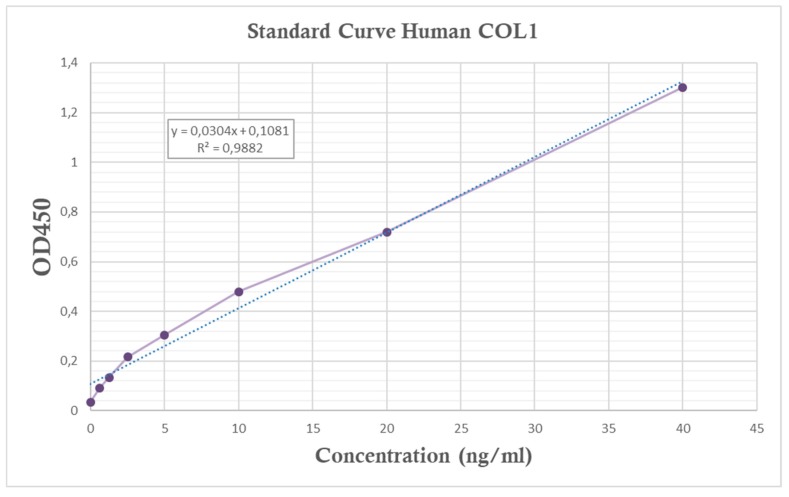
Standard curve used in ELISA assay. In the top-left box, the equation of the line used to obtain the concentrations of the samples is given, in addition to the correlation coefficient R^2^, which indicates a strong relation between the two variables (optical density (OD) and collagen type I (COL1) concentration) as it is close to 1.

After seeding HGFs for 24 and 72 h, the secretion of collagen type I to the culture medium was quantified through an ELISA assay. A standard curve was defined by the absorbance from the standards containing known concentrations of COL1 (Figure 4).

**Figure 5:**
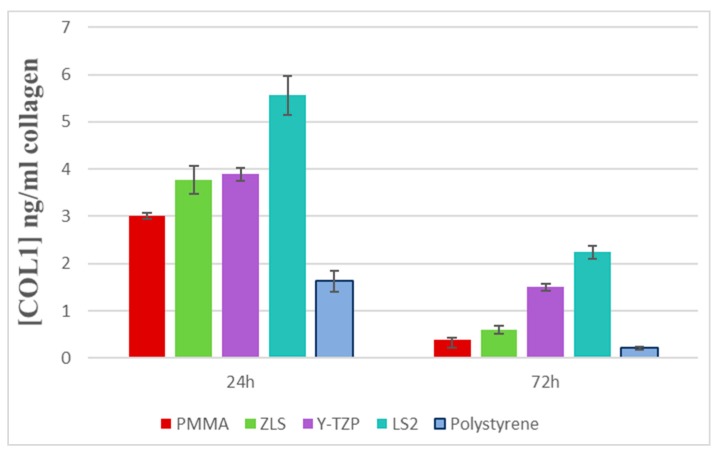
Type I secretion levels at 24 and 72 h.

After 24 h of cultivation, levels of collagen type I showed differences between groups (*p* < 0.01) (Figure 5). LS_2_ (5.56 ± 0.41 ng/mL) showed the highest secretions (*p* < 0.01) compared to the other groups; in decreasing order: Y-TZP (3.89 ± 0.14 ng/mL), ZLS (3.77 ± 0.30 ng/mL), and PMMA (3.01 ± 0.06 ng/mL). The collagen secretion measured in the polystyrene control surface (1.63 ± 0.22 ng/mL) was significantly lower than the other groups (*p* < 0.01). There were statistically significant differences between all groups, except between ZLS–Y-TZP (*p* > 0.05) and ZLS–PMMA (*p* = 0.05).

At 72 h, the levels of secretion of collagen were sharply reduced in all groups (*p* < 0.01 for all groups). The same trend of group distribution was observed. LS_2_ showed the lowest reduction (2.24 ± 0.13 ng/mL), followed by Y-TZP (1.50 ± 0.08 ng/mL), ZLS (0.6 ± 0.08 ng/mL), PMMA (0.38 ± 0.15 ng/mL), and the control group (0.21 ± 0.03 ng/mL). The latter group revealed the highest decrease in collagen secretion. There were significant differences between groups (*p* < 0.01), except for PMMA–ZLS and PMMA–control (*p* > 0.05 for both comparisons).

## 4. Discussion

Biocompatibility refers to a material’s ability to not affect the local or systemic behaviour of an organism. Cytotoxicity is an important biocompatibility component [11,53]. It can be studied in in vivo or in vitro studies. The former has some disadvantages, such as being difficult to control and interpret, in addition to legal and ethical considerations. Nevertheless, the latter offers important advantages: the possibility to study a reaction cell of interest, lower variability results, and easier access to the investigated site [11,51,53,54]. Most in vitro studies of dental material cytotoxicity are cell culture systems [53].

This study investigates the human gingival fibroblasts’ response to the MTT cytotoxicity method and collagen type I secretion on dental CAD/CAM materials, which are widely used as implant crowns and implant transmucosal abutments. Unlike immortal cell lines, primary HGFs keep phenotypically similar features to normal cells, resulting in a very similar imitation to in vivo circumstances [6,49].

Gingiva is the epithelium in charge of creating a barrier (biological seal) between the abutment and the connective tissue. This barrier should adhere to the implant abutment surface, which has the function of creating stability between soft and hard tissues (protecting implant—abutment connection and peri-implant bone), protection against noxious bacteria, and has an acceptable aesthetic quality. The protective barrier requires a nontoxic material that favours the attachment and growth of the surrounding tissues [5,49,54,55,56].

A cell viability assay was conducted with a colorimetric MTT study based on the mitochondrial activity of cells in direct contact with the different surfaces. This kind of research is undertaken in accordance with ISO 10993-5, resulting in a standardised methodology and an objective numeric comparison of the obtained results. Nevertheless, the ISO standard does not have a defined classification to establish cell viability value ranges to determine the grade of cytotoxicity of the materials according to the in vitro-type assay. Publications that do not apply an ISO standard add other variables that complicate the comparison between in vitro studies and the interpreted results [57]. Therefore, conclusions derived from comparisons made with no standardised viability assays should be interpreted with caution.

Most of the studies use an MTT assay, but this is not the only way to analyse cellular viability. One of the methods used was the (2,3-bis(2-methoxy-4-nitro-5-sulfophenyl)-5-[(phenylamino)carbonyl]-2H-tetrazolium hydroxide) (XTT) cytotoxicity test, which used XTT, another salt that produces formazan through mitochondrial dehydrogenases [17,52]. Other similar colorimetric assays are the MTS test and XPS test [6].

MTT is not a new cellular proliferation assay in dentistry. In the year 1993, Li et al. published an experiment about cell reactions to zirconia and other ceramics. Another classic study investigated the cytotoxicity of some metals and ceramics in relation to mouse fibroblasts. They concluded that all ceramic materials had an appropriate biocompatibility [58]. Recently, the biocompatibility of all-ceramic CAD/CAM materials has also been studied [2,25,58,59,60], and the present study contributes to this research.

Our results demonstrated good biocompatibility levels in all the analysed materials. We studied viability levels using a direct-contact MTT assay evaluated at 24, 48, and 72 h periods on three all-ceramic CAD/CAM materials and one polymer-based CAD/CAM material. We determined ascendant viability values over the time points (viability at 72 h > 48 h > 24 h). LS_2_ viability stood out significantly over all the other materials throughout the three-time evaluation points (p < 0.05); however, although some of the differences were statistically significant, Y-TZP and ZLS showed similar viability at 48 and 72 h. The distribution of values was similar in the three studied time lapses. PMMA showed the lowest viability during the three times evaluation points, which also increased over time.

Recently, Atay et al. sought to define a classification where a cell viability above 90% infers the material is not cytotoxic, values between 60–90% are considered slightly cytotoxic, values of 30–59% are moderately cytotoxic, and those below 30% are considered severely cytotoxic [17]. According to this classification, all materials should be considered as moderately cytotoxic at 24 h, all would be considered slightly cytotoxic at 48 h (except PMMA, which would remain moderately cytotoxic), and ZLS and PMMA should be considered as slightly cytotoxic at 72 h. After three days, LS_2_ and Y-TZP should be considered as not cytotoxic. In Atay et al., the cytotoxic evaluation of different CAD/CAM materials was performed using extracts from the culture medium and not by direct contact, so this should be considered.

Lithium disilicate and zirconia are two widely used materials in dentistry because of their good optical and mechanical properties. However, LS_2_ cytotoxicity remains unclear according to the conditions of the study. Some publications have reported the cytotoxicity of this material [24,61], and there is no clear consensus when comparing lithium disilicate and zirconia. A key factor could be the type of cells seeded on the surfaces. In some studies, LS_2_ has been considered cytotoxic for HGFs, categorizing it as a second-class biomaterial [2,6,13,23,28]. In another study, it was observed—despite both materials being biocompatible—that proliferation and viability rates were higher in LS_2_ for epithelial cells [62].

There are many studies comparing zirconia with titanium [49,50,63,64], but only a few have compared CAD/CAM-ceramics with CAD/CAM-polymers. Raffaeli et al. compared the response of rat immortalised fibroblasts to zirconia and feldspathic ceramics, and their findings suggested a better viability in zirconia. In that study, as in ours, they used polystyrene as a control. This material promotes growth and cell adhesion, which makes it widely used for in vitro assays [51]. Similar viability results were obtained in other studies when comparing CAD/CAM zirconia and LS_2_ with feldspathic veneer ceramic, revealing a higher viability for the first two ceramic materials [13].

ZLS is a new material, and there are many publications about the product’s mechanical properties but few about its biocompatibility. In 2017, Dal Pilva et al. conducted a similar study comparing ZLS and Y-TZP cytotoxicity at 24 h. They also reported severe cytotoxicity (<50% cell viability) at early contact [48].

We could only find one study that evaluated CAD/CAM all-ceramic materials and interim prosthesis materials in a proliferation assay. This study revealed a high cell viability (above 90%) for all materials at 24 and 72 h and on the seventh day. Nevertheless, the authors considered zirconia, LS_2_, and Vita CAD-Temp materials as slightly cytotoxic at all incubation periods. This disparity between those results and ours could be explained by the fact that, in the mentioned study, the XTT viability assay was undertaken by an extract method, which could be a potential differentiating factor to consider [17].

One reason for this acute cytotoxicity in the first 24 h has been described by other authors: in the very first hours of the material immersion in the medium culture, ion leaching from ceramics can be expected. This phenomenon is responsible for cytotoxicity to a greater or lesser degree. In our experiment, the two main inorganic elements that are potentially released by LS_2_ and ZLS are alumina and silicon, both of which are considered to have low cytotoxicity [65,66]. Some studies also blame this low viability on Zn, an LS_2_ component considered by some as a cellular-viability suppressor or cytotoxicity increaser [24]. In the case of monolithic zirconia, the leaching elements could be zirconia, yttrium, silicon, and sodium. Nevertheless, polycrystalline ceramic is free from serious corrosion, and the cytotoxic potential of these elements is moderately low, according to the literature reviewed [66,67,68,69].

The most used dental interim materials are made with polymethyl methacrylate, polyethylene methacrylate, or bis-acryl resins. The polymerisation process of these materials produces by-products and unreacted monomers (polymerisation shrinkage) that can be noxious to gingival tissues. When these materials are commercialised in CAD/CAM blocks, they are pre-polymerised, reducing the harmful effects [17,39,45]. However, an incomplete pre-polymerisation may cause the leaching of some of the components of these resin materials [17,70]. The oral environment can lead to the materials’ biodegradation, leaching potentially toxic agents (monomers, in most cases) that affect cells and tissues [39,71]. Degradation takes place as a result of multiple factors (e.g., saliva, bacteria colony, and mastication) [71]. Saliva is fundamentally composed of water, whose molecules can enter the polymers’ structure and give rise to monomer and additive diffusion [17,70,71]. This phenomenon can occur in in vitro conditions as well, due to the aqueous base of the culture medium that can generate similar consequences as those of saliva [71].

Shim et al. compared some provisional materials in relation to the cytotoxic effect of HGFs, and they concluded PMMA and bis-acryl (viability >80%) have much better effects with fibroblasts than poly(ethyl methacrylate). However, Atay et al. considered Vita CAD-Temp to be slightly cytotoxic.

Our PMMA results can be explained if we consider that this is not a definitive material. This polymer can be used as a long-term provisional material, lasting up to two years according to the manufacturer. We cannot expect this polymer to exhibit definitive material behaviour because this kind of product is commercialised with the purpose of conforming the gingival tissue before the definitive material is placed, and also to protect the peri-implant space [38,42]. However, it should be easily removed by a professional without producing a new soft-tissue regenerating period.

In accordance with the obtained results, it cannot be said that these materials are totally inert; nevertheless, the cytotoxic values after 72 h are within the biocompatibility range for clinical use, as other authors have claimed [17,72,73].

After the placement of an implant abutment during the second-stage surgery, the wound must heal through the migration of fibroblasts and the formation of a collagen matrix [55]. To achieve the successful integration of a ceramic material, cells must colonise the surface, and elements of the extracellular matrix (ECM) must be remodelled, such as collagen, which plays an important role in cell adhesion to a surface [13,74].

Many of the published studies measure collagen mRNA expression, along with other cellular adhesion proteins, through reverse transcription polymerase chain reaction (PCR) assays. In these cases, collagen type I mRNA is isolated and extracted from HGFs, cultured on different study materials, and converted to cDNA [50,56,63]. Nevertheless, few publications have evaluated the collagen secretion on ceramic material through enzyme-linked immunoabsorption assays.

Fibroblasts are continually remodelling their ECM conformation because they adhere to a surface. These adjustments are done through the synthesis and proteolysis of the components. In our study, collagen type I secretion to the culture medium was determined in order to evaluate the correct function of fibroblasts. After 24 h, LS_2_ was the surface with the highest level of collagen secretion, with statistically significant differences observed between all groups (p < 0.01), except in ZLS, Y-TZP, and PMMA, which showed similar values (p > 0.05). Collagen secretion decreased sharply for all groups at 72 h, and again, LS_2_ was the surface with the highest value (p < 0.01). At this time evaluation point, there were no significant differences in ZLS, PMMA, and the polystyrene control group. The reason for this drastic secretion decrease has been explained by other publications [13,63].

Tetè et al. compared collagen type I secretion by HGFs at 24 and 72 h on different polished and unpolished ceramic surfaces and a polystyrene control. At 24 h, no significant differences were observed for polished or unpolished zirconia and glazed feldspathic surfaces; however, secretion levels on a polished LS_2_ surface were statistically lower. At 72 h, a sharp decrease was detected in all groups and was statistically significant for both polished zirconia and LS_2_ surfaces [13]. The control surface revealed the lowest secretion levels at both time evaluation points. Some researchers have attributed this to the maximum proliferation levels on polystyrene surfaces at the expense of the synthesis and assembling of the ECM components [75]. They stated that, in normal in vivo conditions, cells show a minimum proliferation rate and high collagen secretion levels, but in in vitro conditions, cellular behaviour is different. In the first hours of in vitro testing, cells showed a greater tendency to secrete collagen to enhance adhesion to the substrate and later on stimulating their proliferation. This could explain why collagen secretion sharply decreases beyond 72 h after cells adhere [13]. When cellular confluence is reached, proliferation stops by inhibition contact, and collagen secretion also decreases drastically after cells anchor to the surface [63].

The low values obtained in our results are due to another cause: collagen secretion differs depending on the type of fibroblast. Gingival fibroblasts express less collagen and present a different ECM organisation regarding periodontal ligament fibroblasts because HGFs have a lower expression of α2β1 and α10β1 integrins [76,77,78,79].

More research is needed to analyse the possible relationship between surface, material type, cell proliferation, and ECM protein secretions, such as collagen. In in vitro studies, factors such as the percentage of supplemented FBS, pH, or the composition of the culture medium are additional variables that can influence the collagen levels secreted by cells [79]. Assays should be undertaken under ISO standards in order to be reproducible and make objective comparisons of the results [6,9,80].

## 5. Conclusions

According to the results obtained and the limitations of the present in vitro study, it may be concluded that ceramic materials—more precisely, lithium disilicate—revealed better cell responses than polymers in terms of cell viability and collagen type I secretion. Regarding direct-contact cytotoxicity, a linear effect of time and type of material was observed with decreased cytotoxicity over time (*p* < 0.05). At 72 h, all groups reached biologically acceptable levels. Lithium disilicate showed the highest levels of collagen type I both at 24 and 72 h. The secretion levels were sharply reduced in all groups at 72 h (*p* < 0.01). Polymers showed poor results on both types of experiments. Nevertheless, further studies are needed to consolidate these findings and thus extrapolate the results into clinical practice.

## Figures and Tables

**Table 1:** The brand names, types, compositions, and manufacturers’ data of the materials.

Specimen	Material Type	Composition	Manufacturer	Lot No.
Vita CAD-Temp^®^ monoColor (PMMA)	Polymethacrylate	C_5_O_2_H_8_, SiO_2_ and pigments	VITA Zahnfabrik, Bad Säckingen, Germany	1M27/51750
Celtra^®^ Duo (ZLS)	Zirconia-reinforced lithium silicate	SiO_2_, Li_2_O, P_2_O_5_, Al_2_O_3_, ZrO_2_, CeO_2_, Tb_2_O_3_	Degudent GmbH, Hanau-Wolfgang, Germany	HT-A1-C14/16002830
IPS e.max^®^ CAD (LS_2_)	Vitreous ceramic lithium disilicate	SiO_2_, Li_2_O, K_2_O, MgO, ZnO, Al_2_O_3_, P_2_O_5_	Ivoclar Vivadent, Schaan, Liechtenstein	HT A1/C1 4 /V28352
VITA YZ^®^ (Y-TZP)	Zirconia partially stabilised with yttrium oxide	Al_2_O_3_, ZrO_2_, Y_2_O_3_, Fe_2_O_3_, Er_2_O_3_, Hf_2_O_3_	VITA Zahnfabrik, Bad Säckingen, Germany	YZ Twhite/74970
